# Tooth extraction education at dental schools across Europe

**DOI:** 10.1038/bdjopen.2015.2

**Published:** 2015-10-23

**Authors:** Henk S Brand, Carlijn C J van der Cammen, Sophie M E Roorda, Jacques A Baart

**Affiliations:** 1 Department of Oral Biochemistry, Academic Centre for Dentistry Amsterdam (ACTA), Amsterdam, The Netherlands; 2 Department of Medical–Dental Interaction, Academic Centre for Dentistry Amsterdam (ACTA), Amsterdam, The Netherlands; 3 Department of Oral and Maxillofacial Surgery, VU University Medical Centre/Academic Centre for Dentistry Amsterdam (ACTA), Amsterdam, The Netherlands

## Abstract

**Objectives/Aims:**

To explore students’ opinion about theoretical and clinical training in tooth extraction at different European dental schools.

**Materials and Methods:**

An online questionnaire, containing 36 dichotomous, multiple choice and Likert scale rating questions, was distributed among students of 56 different dental schools. After excluding schools where <20 students responded, 656 questionnaires from 23 dental schools remained for statistical analysis.

**Results:**

Dental schools showed a wide variation in the initial practical teaching of tooth extraction, from years 2 to 6. Several schools used a preclinical training model, and most students considered this useful. Some students considered their knowledge about forceps and elevators insufficient (6–60%), as well as their preparation for complications (5–60%). Students usually had received education in forceps and elevator techniques. Inclusion of (non)surgical removal of retained roots and surgical removal of third molars showed a wide variety between dental schools. Less than half of the students reported education in surgical removal of impacted teeth. Students from four of the 23 dental schools felt insufficiently prepared in tooth extraction (Likert scale ⩽3).

**Conclusion:**

There is a wide variation among European dental schools in teaching programs of tooth extraction and the rating of these programs by students.

## Introduction

Tooth extractions are frequently performed in the general dental practice. Forceps exodontia of teeth is established as a basic clinical skill for dental graduates. During forceps exodontia, however, there is always the possibility of fracture of parts of the root and the necessity to start a surgical extraction. This means that graduating dentists must be competent in both surgical techniques. This is reflected in the current profile for the European dentist.^1^ Tooth extractions are mentioned in competences 6.53 and 6.54, which state that a dentist must be competent at ‘performing uncomplicated extraction of erupted teeth’ and ‘performing surgery for the uncomplicated removal of fractured or retained roots and the removal of uncomplicated partially erupted teeth’.

Various studies in the UK have evaluated the perceptions of recently graduated dentists about their preparedness to perform extractions in the dental practice. Almost all respondents perceived that the teaching at their dental school had given them sufficient knowledge to undertake simple forceps exodontia, but confidence levels to perform surgical extractions were considerably lower.^2–8^ Similar results were observed for graduates of the dental school of the University of Hong Kong. Eighty-nine per cent of the students felt well prepared to perform simple extractions and 62% felt well prepared to extract impacted third molars.^9^

Surveys among staff and students of dental schools across Europe have shown a considerable variation in dental curricula with regard to the teaching of local anaesthesia, tooth colour determination systems and fixed prosthodontics.^10–13^ This variation in teaching can influence the level of confidence of dental students, which may also apply when administering a tooth extraction in a patient. This suggestion is supported by the observation that students from the dental school in Cardiff in the UK were significantly more confident in performing simple extractions, as well as surgical extractions than students from the dental school in Cork in Ireland.^6^ Therefore, the aim of the present study was to explore the perception of students from different dental schools in Europe about their education in tooth extraction.

## Materials and Methods

This study is part of a series of studies performed by the Academic Centre of Dentistry Amsterdam, which explores the variation in curricula between dental schools across Europe.^10–13^ For the present study, an online questionnaire about the teaching of tooth extraction was developed. The first part of the questionnaire collected general information about dental school, gender and study year. In the second part, the extraction education of the student was explored with 36 dichotomous, multiple choice or rating scale questions. The opinion of the respondents about several aspects of the extraction education was rated with five-point Likert scales. A score of 1 meant ‘absolutely not’ or ‘very bad’ and a score of 5 did mean ‘absolutely’ or ‘very good’. The total number of questions to be answered depended on the student’s individual situation.

The questions were entered in the internet survey program eXamine 2.0.^14^ For the distribution of the questionnaire, the Deans of 145 dental schools who were member of the Association for Dental Education in Europe were approached. In addition, all delegates mentioned on the website of the European Dental Students Association were approached. A web link to the questionnaire was sent with an explanatory E-mail to the Deans and European Dental Students Association delegates, and they were asked to distribute the web link among all students of their dental school. The questionnaire was distributed in October and November 2011. The students were asked to answer the questionnaire within a period of 6 weeks. Participation was on a voluntary base, and all responses were anonymous.

The total number of respondents was 1,294 from 56 different dental schools in Europe. Questionnaires were considered useful when the respondent reported to have received education in tooth extraction. Less than 20 students from dental schools in Aachen, Berlin, Heidelberg, Leipzig (Germany), Clermont, Lille, Lyon, Montpellier, Nancy, Nice, Paris, Reims, Strasbourg (France), Groningen (Netherlands), Bergen (Norway), Arkhangelsk, Chelyabinsk, Kazan, Kirov, Moscow, Omsk, Samara, St Petersburg, Ufa (Russia), Novi Sad (Serbia), Ankara, Istanbul (Turkey), Bristol (UK) and Stockholm (Sweden) returned useful questionnaires. These numbers were considered too small to be representative and these dental schools were excluded from the statistical analysis. An exception was made for the very small dental school in Msida (Malta), where more than half of the students who had received education in tooth extraction returned the questionnaire. This resulted in 656 useful questionnaires from 23 different dental schools remaining for analysis (Table 1).

Data are expressed as percentages or mean±s.d. The rating scale items were statistically analysed using IBM SPSS Statistics version 21.0 (IBM Crop, Armonk, NY, USA). For overall analysis, the Kruskal–Wallis test was used followed by Mann–Whitney tests as *post hoc* procedure for pair-wise comparisons. Potential relations between parameters were explored with Spearman’s rank order correlation coefficient. All levels of significance were set at *P*<0.05.

## Results

Table 1 presents general information about the respondents. In general, the percentage of male students is <50%, and usually they are in the fourth year of the study. The teaching of the theoretical aspects of tooth extraction usually starts in year 3, with Brest, Kosice and Plymouth starting 1 year earlier. The initial teaching of the practical aspects has a much wider variation in the dental curricula, and ranges from year 2 (Plymouth) to 6 (Amsterdam).

Table 2 gives an overview of the study material used during teaching of tooth extraction. Most dental schools use one or more textbooks (18–100%) and handouts (9–96%). Readers (0–73%) and digital video discs or films (0–50%) are less frequently used. In general, students were quite satisfied with the provided material, with scores varying from 2.9 to 4.1 on a 5-point Likert scale.

The use of a preclinical training model before the first extraction in a human is frequently reported by students from Cardiff, Gent, Kosice, Leeds, Nantes, Plymouth and Turku (Table 3). The majority of the students who used a preclinical training model considered it a useful preparation for the subsequent tooth extraction in a patient. However, in Cardiff and Ghent, only small numbers of students found the preclinical training model useful (27% and 36%, respectively).

Students feel relatively well prepared in several areas related to perform a tooth extraction. Some students felt that their knowledge about forceps and elevators was insufficient (6–60%), as well as their preparedness for complications (5–60%). Only few students from all dental schools felt insufficiently prepared with regard to anatomy, prescription of analgesics, medication problems or legal aspects of tooth extraction.

Supervision during the first tooth extraction in a patient was mostly performed by a dentist or oral-maxillofacial surgeon (Table 4). In general, students were quite satisfied with the supervision, with scores varying from 3.6 to 4.8.

There is a wide variation in specific extraction techniques included in the curricula of the surveyed dental schools (Table 5). A large majority of the students report that they have received education in forceps and elevator techniques (42%–100% and 44%–100%, respectively). For non-surgical and surgical removal of retained roots, as well as surgical removal of third molars, much larger differences between dental schools were observed (5%–83%, 6%–71% and 0%–80%, respectively). Only a minority of the students received education in surgical removal of impacted teeth (0–50%). The widest variety in extraction techniques seems to be provided by the dental schools in Copenhagen, Nantes, Szeged and Plymouth.

Finally, Table 6 shows that students of dental schools across Europe vary considerable in their opinion whether they are properly trained in tooth extraction. Students from the dental school in Plymouth felt best prepared, closely followed by students from the dental schools in Szeged, Copenhagen, Trieste, Nantes and Sofia (all mean scores ⩾4). The students from 4 of the 23 surveyed dental schools felt insufficiently trained in tooth extraction (mean scores ⩽3). Students from the dental school in Szeged rated their training the highest (4.5), closely followed by students from the dental schools in Sofia and Plymouth. Students from 5 of the 23 surveyed dental schools were not very satisfied with the education in tooth extraction (mean scores ⩽3). The mean opinion of students about the education at their dental school correlated significantly with the year in the curriculum of the initial teaching of the practical aspects of tooth extraction (*r*=−0.629, *P*=0.001). For the initial theoretical teaching, this relation with the overall satisfaction did not reach significance (*r*=−0.388, *P*=0.067).

## Discussion

The present study of dental students’ perceptions showed considerable variation among European dental schools in the teaching of tooth extraction and the rating of this teaching by students. This is in line with a previous study in the UK, showing variations between dental schools in content and delivery of the oral surgery clinical teaching programs.^15^ The undergraduate teaching of wisdom tooth removal in the UK showed also variation in the stage of the curriculum where this topic is taught.^16^

The initial teaching of the practical aspects of tooth extraction varies considerable between European dental schools with regard to study year (Table 1). The rating of the education at dental schools correlated negatively with the year of the initial teaching of the practical aspects of tooth extraction, indicating that an early clinical exposure is appreciated by the students.

Early exposure may also increase the number of extractions achieved by dental students during their clinical years. In 2008, the minimum number of extractions that undergraduates were expected to achieve during their clinical years varied between 11 dental schools in the UK from 20 to 115.^15^ Shortage of suitable cases for undergraduates^17^ has been suggested to be one of the limits to develop confidence,^6^ as the number of surgical extractions performed increased competence.^18^ However, in another study, no significant relationship was observed between the total number of teeth extracted and the successful completion of the final assessment.^5^ Therefore, during recent years, setting numerical targets has increasingly been replaced in dental school curricula by a competency-based approach, although certain numerical targets are still present in most curricula.^6^

Knowledge of the relevant anatomy is important to perform a tooth extraction correctly. A few dental students in Europe felt insufficiently prepared with regard to anatomical aspects of extractions (Table 7). This is in line with a recent national survey of UK final year dental students, where 78% of the students reported that the anatomy teaching had been appropriate to their clinical needs.^7^ The percentage of students who feels insufficiently prepared for potential complications during a tooth extraction in a patient varied in the present study considerable between dental schools, from 0 to 60%. We did not specify different types of potential complications in our questionnaire. In the UK, a high percentage of the final year dental students feel confident to manage haemorrhage.^7,8^ Therefore, it might be interesting to explore in future studies which (other) complications are anticipated by dental students in Europe.

Preclinical training on manikins may assist dental students to develop operative skills, may increase their level of competence and facilitate the transition to the clinic.^19^ In the UK, several dental school use preclinical models for the teaching of extraction skills. These models include commercially available models, virtual learning environments, pigs’ heads and a rubber dam stretched over a cup.^15,16,18^ The present study shows that preclinical models are used at a considerable number of dental schools in Europe. At dental schools where a preclinical training model is widely used, the students considered it a useful preparation for the subsequent tooth extraction in a patient (Table 3). However, at two dental schools only few students found the preclinical training model useful. These differences in appreciation might be related to the type of preclinical model used and/or the amount of time to practice with it. Supervision during the use of the training model, as well as the amount of time between the training with the model and the transition to the clinic may also affect the opinion of the student. Further studies on the effectiveness of different types of preclinical training models for the teaching of extraction skills seem warranted.

Student feedback is an important component to monitor academic programs. Their input gives insight in teaching effectiveness and allows dental schools to identify possible weaknesses in their curriculum, which can result in improvement of clinical teaching.^7,8^ However, in the present study, the current questionnaire was distributed through representatives of the European Dental Students Association. These students are more interested in education in general,^20^ which could have introduced a certain selection bias in the participating students. Another limitation of the type of web-based survey used in the present study is that a considerable amount of time may have passed between the moment that students received their training on tooth extraction and the moment they filled in the questionnaire, which could have affected the accuracy of the responses. In addition, the questionnaire was distributed to all European dental students in English. As this is not the native language of most students, this potentially could have resulted in misinterpretation of some questions. Finally, the question whether students feel properly trained in tooth extractions did not discriminate between their preparation to perform simple extractions and surgical extractions. Recently graduated dentists usually express the opinion that their dental school had given them sufficient knowledge to undertake simple forceps exodontia, but they feel much less prepared for surgical extractions.^2–9^ Considering the average study year of the respondents in the present study, several students may not have followed the complete study programme with regard to extractions at their dental school. As surgical extractions are taught at a later stage, this means that the expressed opinions of the European dental students will most rely on their experiences with forceps extractions.

Despite these limitations, this study supports previous studies which showed that European dental schools vary considerable in their curriculum.^10–13^ This variation in teaching programs could result in different level of competences of recently graduated dentists from different dental schools.^6^ Considering the international mobility of the contemporary dentists, a drive towards more convergence in dental education in Europe seems warranted.^21^

## Figures and Tables

**Table 1:** General characteristics of the responding students from 23 different European dental schools with regard to gender, study year and the year in which the students received the initial theoretical and practical teaching in extraction of teeth

*Dental school*	*Country*	*Useful questionnaires*^a^	*Male (%)*	*Study year (mean)*	*Initial teaching in year*	
					*Theoretical (mean)*	*Practical (mean)*
Amsterdam	The Netherlands	53	47	4.8	4	6
Bern	Switzerland	20	40	4.3	3	3
Bordeaux	France	49	41	5.2	3	4
Brest	France	21	57	3.8	2	4
Cardiff	United Kingdom	25	24	4.3	3	3
Copenhagen	Denmark	25	24	5.0	4	4
Ghent	Belgium	23	35	5.1	4	4
Kaunas	Lithuania	53	23	4.1	3	4
Kosice	Slovenia	21	43	4.8	2	4
Leeds	United Kingdom	23	52	4.5	3	3
London	United Kingdom	32	38	4.7	3	3
Msida	Malta	12	50	4.3	3	4
Nantes	France	23	57	4.3	3	3
Nijmegen	The Netherlands	32	22	4.8	3	4
Oslo	Norway	21	38	4.5	4	5
Plymouth	United Kingdom	21	62	3.5	2	2
Rennes	France	23	48	4.8	3	4
Sofia	Bulgaria	54	41	4.7	3	3
Szeged	Hungary	25	40	4.4	3	3
Toulouse	France	27	56	5.2	3	4
Trieste	Italy	25	44	4.4	3	3
Turku	Finland	23	22	4.3	3	3
Ulm	Germany	24	38	4.6	3	4

a Questionnaires were considered useful when the respondent reported to have received education in tooth extraction and completed at least half of the questions. When 20 or more questionnaires from a dental school were returned or more than half of the students had returned the questionnaire the dental school was included in the analysis.

**Table 2:** Material used during teaching of tooth extraction at 23 different European dental schools and the opinion of the students about the material provided (range 1 = absolutely not to 5 = absolutely)

*Dental school*	*Book(s)*	*Reader*	*Handouts*	*Film/DVD*	*Other/none*	*Satisfied (range 1–5)*
Amsterdam	86	22	76	4	10	3.4±1.0
Bern	40	25	95	15	5	4.0±0.7^a^
Bordeaux	81	42	22	25	3	3.6±0.9^b^
Brest	100	38	25	6	0	3.3±0.8^b^
Cardiff	57	17	70	13	30	2.9±1.3^b,c^
Copenhagen	100	14	73	23	18	3.4±0.9^b^
Ghent	18	9	77	5	18	3.1±1.0^b^
Kaunas	100	22	49	31	10	3.5±0.9^b^
Kosice	100	10	50	15	15	2.9±0.8^b,c,h^
Leeds	57	17	96	13	30	3.6±0.9^e,i^
London	69	7	90	17	20	3.6±0.9^i^
Msida	100	0	9	0	0	3.2±0.9^b^
Nantes	59	73	50	32	14	4.0±0.8^a,d,e,g,h,i,l^
Nijmegen	100	31	35	3	17	3.5±0.8^b,i^
Oslo	56	0	56	0	69	2.9±1.1^b,c,m^
Plymouth	80	20	80	50	20	4.1±0.9^a,c,d,e,f,g,h,i,j,k,l,n,o^
Rennes	65	40	20	50	5	3.7±0.8^e,i^
Sofia	98	18	18	32	2	4.1±0.8^a,c,d,e,f,g,h,i,k,l,n,o^
Szeged	96	9	78	13	4	4.1±1.0^a,c,d,e,f,g,h,i,k,l,n,o^
Toulouse	44	39	44	30	9	3.5±0.7^b,i,m,p,r,s^
Trieste	96	12	52	24	8	4.0±0.7^a,d,e,f,g,h,i,l,n,o,t^
Turku	65	13	87	22	4	4.0±0.8^a,d,e,g,h,i,l,o,t^
Ulm	39	39	35	39	17	2.9±0.5^a,b,c,f,h,j,k,m,n,p,q,r,s,t,u,v^

Data are expressed as percentages or mean score±s.d.

*P*<0.05.

Abbreviation: DVD, digital video disc.

^a^versus Amsterdam, ^b^versus Bern, ^c^versus Bordeaux, ^d^versus Brest, ^e^versus Cardiff, ^f^versus Copenhagen, ^g^versus Gent, ^h^versus Kaunas, ^i^versus Kosice, ^j^versus Leeds, ^k^versus London, ^l^versus Msida, ^m^versus Nantes, ^n^versus Nijmegen, ^o^versus Oslo, ^p^versus Plymouth, ^q^versus Rennes, ^r^versus Sofia, ^s^versus Szeged, ^t^versus Toulouse, ^u^versus Trieste, ^v^versus Turku.

**Table 3:** The percentage of students from 23 different European dental schools reporting the use of a preclinical training model before their first tooth extraction in a human, and the opinion of the students who used such a model whether it was a useful preparation for the first extraction in a patient

*Dental school*	*Use of training model*	*Useful preparation*
Amsterdam	4	0
Bern	5	100
Bordeaux	21	83
Brest	0	—
Cardiff	68	27
Copenhagen	5	100
Ghent	60	36
Kaunas	10	100
Kosice	67	75
Leeds	68	80
London	7	100
Msida	0	—
Nantes	50	100
Nijmegen	42	70
Oslo	17	50
Plymouth	95	90
Rennes	5	100
Sofia	52	89
Szeged	44	90
Toulouse	21	75
Trieste	4	100
Turku	82	67
Ulm	100	77

Data are expressed as percentages.

**Table 4:** Supervision of students from 23 different European dental schools during their first tooth extraction, the background of the supervisor and the opinion of the students about the supervision (range 1 = absolutely not to 5 = absolutely)

*Dental school*	*No supervision*	*Dentist*	*Oral-maxillofacial surgeon*	*Other*	*Satisfied with supervision (range 1–5)*
Amsterdam	7	56	33	4	3.8±1.2
Bern	0	90	10	0	4.6±0.5^a^
Bordeaux	7	79	4	11	4.1±1.2
Brest	0	100	0	0	4.3±0.9
Cardiff	9	55	18	18	3.9±1.2
Copenhagen	0	70	30	0	4.2±0.9
Ghent	21	52	21	5	3.9±1.0^b^
Kaunas	5	3	76	16	4.1±1.0
Kosice	6	11	83	0	4.4±0.9
Leeds	0	55	36	9	4.4±0.8
London	0	62	34	3	4.0±1.1
Msida	0	90	10	0	4.7±0.7^a,g^
Nantes	29	67	5	0	4.5±0.8^a,g^
Nijmegen	0	35	65	0	4.1±1.1
Oslo	0	92	8	0	4.3±0.9
Plymouth	0	100	0	0	4.5±0.8^a,g^
Rennes	7	79	7	7	4.3±0.8
Sofia	0	18	82	0	4.5±0.9^a,e,g,h,k^
Szeged	0	36	64	0	4.7±0.5^a,c,e,f,g,h,k,n^
Toulouse	0	90	5	5	4.1±1.1^s^
Trieste	24	64	0	12	3.6±1.0^b,i,j,l,m,n,p,q,r^
Turku	0	19	67	14	4.8±0.4^a,c,e,f,g,h,k,n,t,u^
Ulm	0	12	88	0	4.5±0.6^a,g,u^

Data are expressed as percentage or mean score±s.d. For explanation of superscripts, see legend of Table 2.

**Table 5:** Percentage of students from 23 different European dental schools who reported to have received education in specific extraction techniques

*Dental school*	*Forceps techniques*	*Elevator techniques*	*Non-surgical removal of retained roots*	*Surgical removal of retained roots*	*Surgical removal of third molars*	*Surgical removal impacted teeth*
Amsterdam	82	59	48	7	16	0
Bern	70	60	30	20	20	15
Bordeaux	61	71	54	71	39	11
Brest	63	75	35	69	75	13
Cardiff	86	64	5	14	14	0
Copenhagen	70	90	45	70	80	40
Ghent	95	90	42	11	5	5
Kaunas	73	83	27	14	19	8
Kosice	78	44	44	6	6	6
Leeds	100	73	41	36	41	18
London	100	97	83	69	21	17
Msida	100	100	20	10	0	0
Nantes	62	81	52	67	71	38
Nijmegen	87	87	44	13	13	13
Oslo	67	75	33	17	42	17
Plymouth	95	65	55	65	55	40
Rennes	29	71	43	50	50	14
Sofia	72	84	34	36	36	18
Szeged	86	55	68	64	73	50
Toulouse	42	74	47	42	42	11
Trieste	96	80	72	48	28	8
Turku	86	76	47	24	38	10
Ulm	71	59	24	24	24	12

Data are expressed as percentages.

**Table 6:** The opinion of students from 23 different European dental schools whether they feel properly trained in tooth extraction and the overall rating of their extraction education (range 1 = absolutely not to 5 = absolutely)

*Dental school*	*Properly trained (range 1–5)*	*Overall rating (range 1–5)*
Amsterdam	2.8±1.2	2.5±1.0
Bern	3.6±0.7^a^	4.0±0.8^a^
Bordeaux	3.6±0.8^a^	3.8±0.7^a^
Brest	3.6±0.8	4.0±0.8^a^
Cardiff	3.1±1.1^c^	2.9±1.0^b,c,d^
Copenhagen	4.1±0.7^a,b,e^	4.3±0.7^a,c,e^
Ghent	2.7±1.1^b,c,d,f^	3.0±0.9^b,c,d,f^
Kaunas	3.1±1.0^c,f^	3.6±0.9^a,e,f,g^
Kosice	3.5±1.1	3.7±0.9^a,e,f,g^
Leeds	3.8±0.8^a,e,g,h^	4.1±0.8^a,e,g,h^
London	3.9±0.9^a,e,g,h^	4.0±1.0^a,e,g^
Msida	3.4±0.7	3.7±1.0^a^
Nantes	4.0±0.8^a,e,g,h^	4.1±0.8^a,e,g^
Nijmegen	3.3±1.0^f,k,m^	3.3±1.0^a,b,f,j,k,m^
Oslo	2.9±1.2^f,k,m^	3.0±0.8^b,c,d,f,j,k,m^
Plymouth	4.3±0.8^a,b,c,d,e,g,h,i,j,l,n,o^	4.3±0.8^a,c,e,g,h,i,n,o^
Rennes	3.7±0.9^g^	3.8±0.9^a,e,g^
Sofia	4.0±0.7^a,e,g,h,n,o^	4.4±0.6^a,c,e,g,h,i,l,n,o^
Szeged	4.2±0.9^a,e,g,h,n,o^	4.5±0.8^a,c,e,g,h,i,n,o^
Toulouse	3.6±1.1^g^	3.6±0.8^a,e,f,p,r,s^
Trieste	4.1±0.7^a,b,e,g,h,n,o^	4.1±0.7^a,e,g,n,o^
Turku	3.4±1.1^p,s,u^	4.1±1.1^a,e,g,n,o^
Ulm	2.2±1.1^b,c,d,e,f,h,i,j,k,l,m,n,p,q,r,s,t,u,v^	2.8±1.0^b,c,d,f,h,i,j,k,m,p,q,r,s,t,u,v^

Data are expressed as mean scores±s.d. For explanation of superscripts, see legend of Table 2.

**Table 7:** Areas in which students from 23 different European dental schools felt insufficiently prepared before they performed their first tooth extraction in a patient

*Dental school*	*Anatomy*	*Knowledge of forceps and elevators*	*Prescription of analgesics*	*Medication problems*	*Complications*	*Legal aspects*
Amsterdam	0	52	11	19	44	11
Bern	0	10	0	5	0	5
Bordeaux	11	25	18	11	21	11
Brest	19	38	6	13	31	0
Cardiff	14	55	36	32	27	32
Copenhagen	0	30	5	5	25	5
Ghent	5	15	20	10	30	10
Msida	10	60	50	40	60	20
Nantes	0	14	0	5	5	1
Nijmegen	0	25	4	17	42	0
Kaunas	12	20	15	17	34	5
Kosice	22	11	6	22	17	6
Leeds	5	32	0	5	27	0
London	7	45	17	10	38	28
Oslo	25	50	0	8	17	0
Plymouth	5	15	5	0	15	0
Rennes	0	18	12	12	12	12
Sofia	16	6	2	10	20	8
Szeged	13	22	22	22	17	13
Toulouse	32	42	16	16	37	26
Trieste	4	8	4	12	8	0
Turku	9	23	9	23	23	9
Ulm	12	53	0	24	41	6

Data are expressed as percentages.
